# Unmet psychological needs underlying mobile phone dependence among medical undergraduates: a qualitative study based on the basic psychological needs theory

**DOI:** 10.3389/fpsyg.2025.1608607

**Published:** 2025-06-24

**Authors:** Jun Chen, Yanxia Guo, Ruiyu Huang, Gang Liu, Baolu Zhang

**Affiliations:** ^1^Department of Nursing, The Affiliated Hospital, Southwest Medical University, Luzhou, China; ^2^School of Nursing, Southwest Medical University, Luzhou, China; ^3^Faculty of Nursing and Midwifery, Jiangsu College of Nursing, Huaian, China; ^4^School of Continuing Education, Guiyang Healthcare Vocational University, Guiyang, China; ^5^Department of Orthopedics and Center for Orthopedic Diseases Research, Affiliated Traditional Chinese Medicine Hospital of Southwest Medical University, Luzhou, China

**Keywords:** mobile phone dependence, medical students, unmet basic psychological needs, basic psychological needs theory, qualitative research

## Abstract

**Background:**

Mobile phone dependence is recognized as a global public health concern, particularly among medical students. The unmet psychological needs reflected in mobile phone dependence among undergraduate medical students remain unclear. Our study aimed to explore the unmet psychological needs underlying mobile phone dependence among medical undergraduates from the Basic Psychological Needs Theory (BPNT) perspective through interviews.

**Methods:**

Fifteen undergraduate medical students exhibiting mobile phone dependence were recruited through purposive sampling for in-depth, semi-structured interviews at a medical university in China. Data were analyzed using Braun and Clarke’s deductive thematic analysis method.

**Results:**

Our study identified four main themes and nine subthemes. The four main themes are (1) autonomy, which includes low self-regulation, reality pressure evasion, and negative affect; (2) competence, which includes achievement through mobile applications and effects on social performance abilities; and (3) relatedness, which includes social network maintenance and digital medical identity formation, (4) meaning in life, which includes life direction confusion and value confusion. Notably, 46.7% of participants identified relatedness needs as the dominant factor in their mobile phone dependence.

**Conclusion:**

Our study uniquely found that mobile phones serve as tools for medical students to compensate for unmet basic psychological needs, manifested as pressure evasion, achievement-seeking, and professional identity construction, while also revealing meaning in life that extends beyond the BPNT frameworks. Our study recommends that medical schools provide autonomy-supportive environments, create opportunities for skill verification through EPA-based assessments and simulations, and establish mentoring systems with digital platforms for professional development. School psychologists and counselors should implement evidence-based interventions like mindfulness training and group self-regulation programs to foster healthy mobile phone usage.

## 1 Introduction

Mobile phone dependence, also referred to as smartphone addiction or problematic smartphone use in various studies, has emerged as a global public health concern. It is characterized by excessive reliance on mobile phones, leading to impairments in physical and mental health as well as social functioning (Cerutti et al., 2021; Montag et al., 2021; Park, 2005). Studies have shown that the overuse of smartphones can impact individual well-being and may result in decreased learning efficiency and social barriers (Zheng et al., 2014; Lepp et al., 2014; Elhai et al., 2016). Among university students worldwide, smartphone addiction is inversely related to academic performance, with heavy users spending less time on academics, hobbies, and investments (Amez and Baert, 2020). Medical students, as future healthcare professionals, present a unique challenge. Medical education is demanding, with numerous courses requiring substantial scientific knowledge and practical skills, necessitating greater investment of time and effort from medical students (Frenk et al., 2010; Dyrbye et al., 2006).

Previous studies have explored the behavioral manifestations of mobile phone dependence and its associated factors. Studies reveal that medical students spend significant time daily on smartphones, and a large proportion engage in smartphone use during educational activities, with 96.8% using devices during lectures and classes (Clavier et al., 2024; Loredo e Silva et al., 2018). Previous studies have indicated a smartphone addiction prevalence rate of 29.8% among medical students in China (Chen et al., 2017). A systematic literature review on modern teaching technologies in medical education indicates that smart devices have become indispensable tools for medical students’ learning, but also bring risks of overuse (Masters et al., 2016). Furthermore, a review on health professions education during COVID-19 found that mobile devices played a crucial role in remote medical education, while potentially intensifying students’ dependence on these devices (Naciri et al., 2021). Medical students’ increased smartphone dependence results from academic pressure, reliance on social media for peer support, and using smartphones as a maladaptive stress-avoidance mechanism (Pinilla et al., 2015; Verma et al., 2023; Wang et al., 2021). These factors, which are predominantly psychological, reflect unmet psychological needs, which diminish students’ stress adaptability, intrinsic learning motivation, social support, and sense of belonging (Neufeld and Malin, 2019; Zhao et al., 2023; Cheng et al., 2022). Notably, these unmet psychological needs play a crucial role in the development of mobile phone dependence. A previous study has demonstrated that individuals are more prone to developing excessive use behavior when their basic psychological needs are inadequately met in real life, yet highly met in digital media (Allen and Anderson, 2018). Meanwhile, the state of psychological needs during media use experiences is closely associated with users’ media usage patterns and mental health (Gmiro, 2024). Additionally, a study has indicated a substantial negative correlation between basic psychological needs and smartphone addiction, suggesting that when individuals’ basic psychological needs are not adequately met, the risk of smartphone addiction increases (Kwon and Lee, 2017). Evidently, mobile phone dependency is prevalent among medical students and is closely associated with unmet psychological needs.

Although these correlations between unmet psychological needs and mobile phone dependency have been established, the specific manifestations and characteristics of this relationship among medical students as a unique population remain unclear. A previous study on smartphone addiction has predominantly employed quantitative research designs for data collection and analysis. A systematic review of longitudinal studies on smartphone addiction identified various predictive factors, including psychological factors, social environmental factors, and unmet psychological needs (Crowhurst and Yau, 2024). Another systematic review of smartphone addiction among Korean university students found that previous studies mainly recorded a variety of adverse outcomes such as psychological distress, decreased academic performance, and sleep quality issues through various standardized scales and questionnaires (Achangwa et al., 2022). However, these studies struggle to deeply reveal how individuals subjectively experience the process of unmet psychological needs and the complex relationship between these experiences and mobile phone-dependent behaviors. The BPNT, which emphasizes the fundamental role of autonomy, competence, and relatedness in mental health (Deci and Ryan, 2000; Vansteenkiste et al., 2020; Ryan and Deci, 2000), provides a robust theoretical perspective to address these issues. However, the unmet psychological needs reflected in the mobile phone-dependent behaviors of medical students remain unclear. Therefore, our study aims to investigate the unmet psychological needs underlying smartphone dependency among undergraduate medical students through qualitative research methods from the BPNT perspective.

## 2 Methods

### 2.1 Study design

Our study employed a qualitative research design, which is particularly suitable for exploring unmet psychological needs among medical students, allowing us to more comprehensively capture individuals’ subjective experiences and feelings. The qualitative research design enables us to capture subjective experiences and contextual details that quantitative methods struggle to fully present, allowing in-depth exploration of the unmet basic psychological needs reflected in medical students’ mobile phone-dependent behaviors. Although BPNT provides a structured framework, the qualitative research design reveals the unique manifestations and pathways to meet these basic psychological needs within the medical education environment. The research team conducted individual semi-structured in-depth interviews with medical students from various academic years and specializations, with participants meeting mobile phone dependency screening criteria. All interviews were audio-recorded, transcribed verbatim, and analyzed using Braun and Clarke’s deductive thematic analysis method. This analytical approach offered substantial flexibility and enabled a comprehensive understanding of complex data, making it particularly suitable for exploring variations in smartphone usage patterns among medical students with different levels of psychological need satisfaction. The study focused on examining the associative patterns between the three basic psychological needs, including autonomy, competence, and relatedness, and mobile phone-dependent behaviors. To ensure the transparency, systematicity, and comprehensiveness of qualitative research reporting, our study strictly adhered to the Consolidated Criteria for Reporting Qualitative Research (COREQ) checklist (Tong et al., 2007).

### 2.2 BPNT

**Figure 1:**
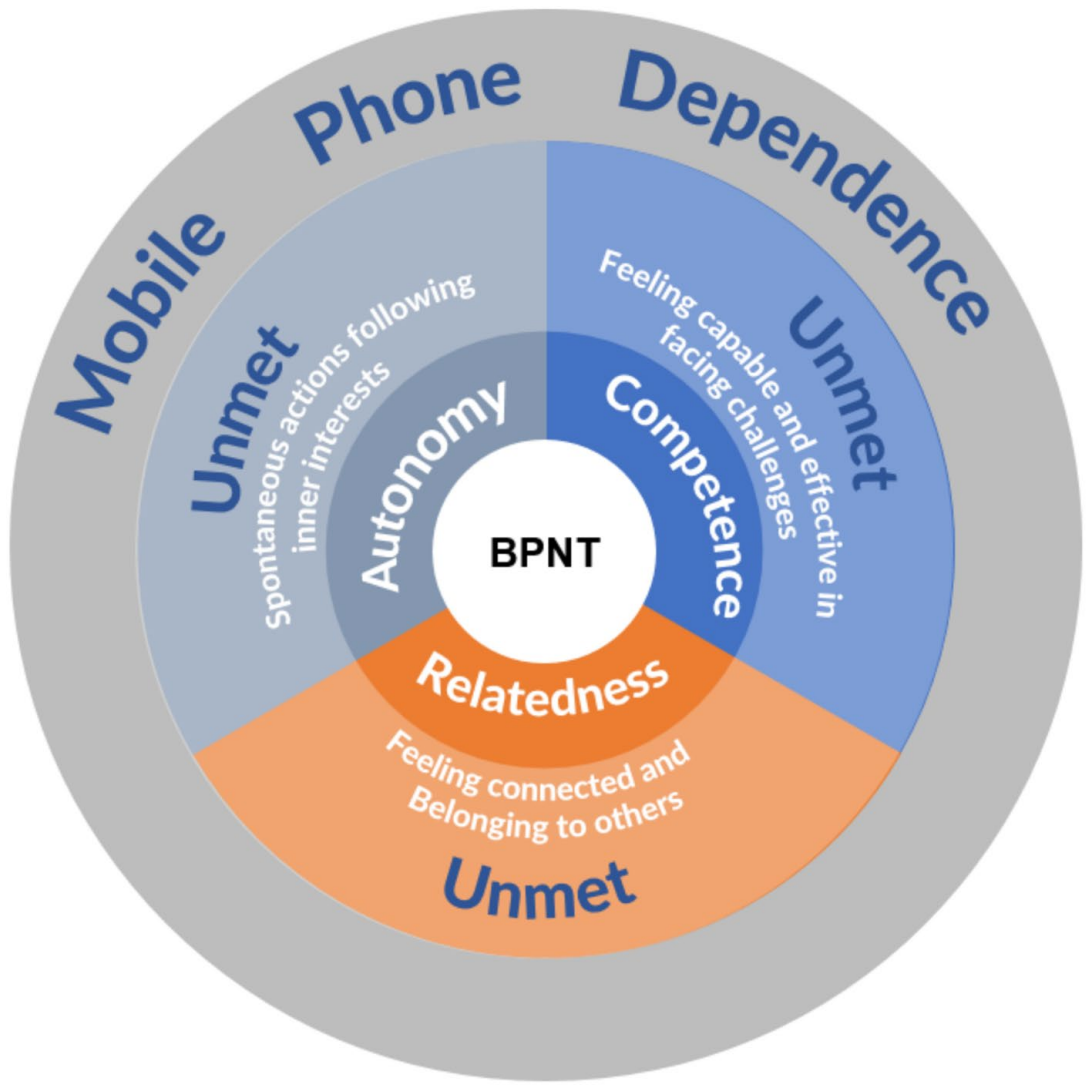
From the BPNT perspective unmet basic psychological needs and mobile phone dependence.

Our study employed the BPNT, a widely recognized psychological motivation theory, as its guiding framework. The BPNT proposes that humans have three innate basic psychological needs: autonomy, competence, and relatedness. The satisfaction of these needs is directly linked to an individual’s psychological well-being and optimal development. These three needs are interwoven, forming a dynamic psychological needs system. When these needs are not adequately met, individuals experience internal psychological imbalance, leading to a persistent sense of discomfort. Specifically, autonomy needs manifest as a desire for personal choice and life control; competence needs reflect an intrinsic motivation to demonstrate and develop skills; relatedness needs point to a basic longing to establish meaningful social connections and a sense of belonging (Deci and Ryan, 2000; Vansteenkiste et al., 2020; Ryan and Deci, 2000). When individuals’ needs remain unmet in a particular domain, they often seek alternative ways to satisfy these needs, with mobile phone dependence emerging as one such compensatory mechanism (Figure 1).

Based on the BPNT, we designed semi-structured interview guidelines focusing on autonomy, competence, and relatedness. During the thematic analysis phase, we used the BPNT theoretical framework as a guide to systematically identify and extract themes related to these three basic psychological needs.

### 2.3 Participants

Our study employed purposive sampling to recruit participants from a medical university in China. First, recruitment information and the Mobile Phone Addiction Tendency Scale (MPATS) questionnaire link were disseminated through student group chats with assistance from counselors of various medical specialties. The questionnaire began with detailed study information and informed consent, which students were required to read and agree to before proceeding. During initial recruitment, researchers did not directly contact potential participants, ensuring the principle of voluntary participation. Second, we selected students with MPATS scale scores ≥48 as potential participants. Third, from these participants, we reached out to those who had voluntarily indicated a willingness for further contact and provided them with additional study details and a minimum 24-h reflection period. Follow-up confirmation of participation was conducted via telephone. Following the principle of data saturation, we ceased sample recruitment when new interviews no longer generated substantive new themes or insights, ultimately determining a final sample size of 15 participants.

The inclusion criteria were: (1) Current undergraduate medical students at Southwest Medical University; (2) MPATS scale score ≥48, indicating potentially high smartphone dependency; (3) Age 18 or above; (4) Voluntary participation with verbal informed consent. The exclusion criteria were: (1) History of severe physical illness or mental disorders; (2) Experience of major life events within the past 3 months that might affect psychological state; (3) Unwilling or unable to complete the full interview process.

### 2.4 Data collection

Our study employed the MPATS scale to screen medical students with mobile phone dependence as research participants (Xiong et al., 2012). This scale has been widely applied and validated in multiple studies (Guo et al., 2022; Li et al., 2019), consisting of 16 items across four dimensions: withdrawal behavior, salience behavior, social comfort, and mood changes. Items are scored on a 5-point Likert scale (1 = strongly disagree to 5 = strongly agree), with total scores ranging from 16 to 80. Considering our qualitative research design and small sample size (*N* = 15), we directly relied on previously validated psychometric properties of the instrument, which demonstrated a Cronbach’s alpha of 0.92 and test–retest reliability of *r* = 0.796.

Referencing the grading methodology of the Mobile Phone Addiction Index (Leung, 2008), and considering the characteristics of the MPATS scale, we classified MPATS scores as follows: mild dependency (48–58 points), moderate dependency (59–69 points), and severe dependency (70–80 points). According to the recommendations of the original scale developers, MPATS scores ≥48 indicate potential mobile phone dependency, while scores <48 suggest rational and moderate usage.

Our study began by using the MPATS for initial screening, selecting medical undergraduates with scores ≥48 as potential research subjects, as this threshold indicates possible mobile phone dependency tendencies. Then, we contacted these students by telephone, explaining in detail the research purpose, methods, and confidentiality measures, while clearly informing them of their right to withdraw from the study at any time without any negative consequences. After obtaining verbal informed consent from the interviewees, a researcher trained in psychology, interpersonal communication, and qualitative research conducted semi-structured, in-depth telephone interviews, ensuring that both parties were in private, quiet environments during the interview to guarantee data quality and participant privacy. The use of a single interviewer helped minimize bias and improve data objectivity. All interviews were audio-recorded, subsequently transcribed verbatim, and cross-checked with the recordings to ensure accuracy. The interview process continued until data saturation was attained, when further data collection produced no novel themes or conceptual insights. During the interviews, participants were invited to provide information about (i) Autonomy, (ii) Competence, and (iii) Relatedness. Although the interview design referenced the three core concepts of BPNT, the questions maintained openness and non-directiveness to ensure authentic capture of participants’ lived experiences. The interview guide included the following questions:

1. Could you describe your mobile phone usage patterns in everyday life?
2. How do you control your mobile phone use?
3. How does your mobile phone influence your ability and confidence to complete tasks and overcome challenges?
4. How does mobile phone use affect your communication and relationships?

### 2.5 Data analysis

Our study employed Braun and Clarke’s deductive thematic analysis framework (Braun and Clarke, 2006), systematically integrated with the BPNT. Firstly, all recorded interviews were transcribed verbatim, and two researchers repeatedly read the transcripts and made initial observational notes, capturing preliminary insights and patterns to achieve data familiarization. Researchers focused on open observation and reading during this phase, approaching the data without any theoretical perspective, concentrating on the content presented in the data itself and participants’ original expressions, ensuring a comprehensive capture of participants’ authentic experiences. Secondly, the two researchers independently conducted line-by-line systematic coding of the transcripts, focusing on patterns and meanings naturally emerging from the data. Subsequently, they compared their initial codes, identifying areas of overlap while forming and continuously refining the coding scheme; if disagreements arose during the coding process, a third researcher was designated to review and resolve the inconsistencies, implementing investigator triangulation to enhance the credibility and reliability of the analysis (Denzin, 2017). Thirdly, potential themes were formed by systematically grouping related codes, allowing themes to emerge naturally from the data. Subsequently, we found that the themes identified in the data had considerable alignment with the BPNT framework. Accordingly, we utilized the conceptual language of BPNT to further refine the themes, ensuring that theme labels accurately captured the complex relationship between mobile phone dependence behaviors and unmet basic psychological needs, while remaining faithful to the original meaning of the data. Fourthly, a theme review was conducted through a two-level process that involved examining the coherence of coded data within themes and evaluating individual themes’ relationship to the entire dataset and research questions, thereby constructing a thematic network framework that is grounded in the original data while incorporating perspectives from the theoretical framework. Finally, we proceeded with further defining and refining theme names, with the final theme definitions and naming considering both the language of the original data and drawing on BPNT theoretical terminology, forming an organic integration of theory and data.

### 2.6 Ethical considerations

Our study was approved by the Biomedical Ethics Committee of Southwest Medical University (SWMUIRBTX-2024100034). Given the minimal risk and online recruitment, verbal informed consent was authorized and obtained via phone after participants were fully informed of the study and their rights. All consent was documented.

## 3 Results

Fifteen undergraduate medical students, aged 18–22, participated in our study. They represented four medical specialties, with MPATS scores at or above 48 points. Based on the MPATS scoring criteria, 9 participants exhibited mild dependency, 3 showed moderate dependency, and 3 demonstrated severe dependency. The mean MPATS score across all participants was 59.2, indicating a moderate level of mobile phone dependence in the sample. The final sample comprised 4 males and 11 females, with 46.6% of participants from nursing programs. This gender distribution is consistent with the characteristics of China’s healthcare system, where nursing is predominantly female (Li et al., 2024). The interviews conducted with participants lasted between 30 and 45 min. Table 1 presents an overview of participant characteristics.

**Table 1:** Demographic characteristics of participants (*N* = 15).

P	Age	Major	Gender	MPATS scores
P1	20	Nursing	Female	58
P2	20	Nursing	Female	56
P3	18	Nursing	Female	64
P4	20	Clinical Medicine	Female	67
P5	21	Clinical Medicine	Male	48
P6	19	Pediatrics	Female	65
P7	22	Clinical Medicine	Male	48
P8	21	Nursing	Female	49
P9	20	Clinical Medicine	Female	73
P10	21	Clinical Medicine Program in Traditional Chinese and Western Medicine	Male	72
P11	21	Pediatrics	Female	58
P12	20	Nursing	Female	72
P13	22	Nursing	Male	52
P14	21	Clinical Medicine	Female	48
P15	20	Nursing	Female	58

48–58 points: mild dependency, 59–69 points: moderate dependency, 70–80 points: severe dependency.

Through analysis of participant interviews, our study identified four main themes and nine subthemes. These four themes are (i) Autonomy, (ii) Competence, (iii) Relatedness, and (iv) Meaning in life. Among these, the majority of participants (*n* = 7, 46.7%) identified relatedness as the primary factor in their mobile phone dependence, followed by autonomy (*n* = 3, 20%), competence (*n* = 3, 20.0%), and meaning in life (*n* = 2, 13.3%). Table 2 provides a comprehensive overview of themes, subthemes, and categories.

**Table 2:** Themes, subthemes and categories obtained from students.

Themes	Subthemes	Categories
Autonomy (*N* = 3, 20%)	Low self-regulation	Habitual consumption
		Excessive use
		Poor temporal control
		Attention deficit
	Reality pressure evasion	High levels of academic stress
		Recreational pursuits
	Negative affect	Guilt
		Anxiety
Competence (*N* = 3, 20%)	Sense of achievement	Digital games
		Skill acquisition
	Social competence impairment	Social skill degradation
		Limited expression abilities
Relatedness (*N* = 7, 46.7%)	Social relationship maintenance	Preserving friendship and family ties
		Teacher guidance
	Digital medical identity formation	Medical information acquisition and sharing
		Expansion of professional digital networks
Meaning in life (*N* = 2, 13.3%)	Life direction confusion	Life emptiness
		Lack of purpose
	Value confusion	Spiritual pursuit deficiency
		Value disorientation

### 3.1 Autonomy

#### 3.1.1 Low self-regulation

Medical students frequently reported unconscious and automatic phone use that had become deeply habitual. Participants exhibited extensive mobile phone engagement that far exceeded moderate usage levels, with many reporting excessive and prolonged daily screen interactions that dominated their waking hours. Moreover, they described significant difficulty in controlling usage duration and setting boundaries for when to start and stop using their devices.

*“I spend a lot of time on my phone, about 8 h daily. While I do not use it during classes, work, or when it’s inappropriate, I often catch myself mindlessly using my phone whenever I have free time. My self-control with phone usage is not great – especially when I’m bored, I automatically reach for my phone.”* (P1)

*“In my daily life, I’m almost constantly on my phone unless it runs out of battery or I’m specifically asked not to use it… Due to my poor self-control, I’ve never actively tried to limit my phone usage. If circumstances allow, I’ll keep using it. I spend approximately 8–9 h on my phone each day.”* (P2)

*“I use my phone for about 8 h every day… My self-control is quite poor, and I can only control it a little… I’m quite addicted to my phone and have trouble controlling how much time I spend on it, but I’m trying my best to control it… My phone dependency tends to distract me.”* (P3)

*“Whenever the teacher’s lecture slightly deviates from the main topic or shifts to a more casual conversational tone, I unconsciously reach for my phone to check messages or browse social media and entertainment content. This instinctive behavior not only diverts my attention but also compromises my focus on and absorption of the course material.”* (P5)

*“My phone usage habits are quite frequent, essentially beginning from the moment I wake up in the morning and continuing throughout my daily activities. On average, I spend approximately seven to eight hours on my phone each day.”* (P12)

*“I face significant challenges in controlling my phone usage, as I often find it difficult to resist the allure of my device. Even during classes, I involuntarily feel the urge to check my phone, with this temptation pervading my entire learning experience.”* (P15)

#### 3.1.2 Reality pressure evasion

Medical students face substantial learning demands and intense pressure, with their phones serving as tools for stress avoidance. Participants regularly used their phones to listen to music, watch videos, play games, and engage in other entertainment activities to relax and temporarily escape academic pressures, particularly during exams.

*“When using my phone, my primary activities involve browsing short video platforms like TikTok, as well as watching* var*ious television shows and videos. These entertainment forms occupy the majority of my screen time.”* (P1)

*“My phone use significantly affects my autonomy, especially during final exam preparation. When I’m in the study room, I unconsciously want to check my phone when the pressure gets high. I tell myself I’ll play with it for a moment, but that ‘moment’ often turns into an unknown amount of time.”* (P4)

*“My phone seriously impacts my independence, especially my study efficiency. After studying for so long, I keep thinking I deserve a break to play on my phone, using it to escape academic pressure.”* (P6)

*“When I’m under stress, I desperately seek ways to relax, and my phone becomes my go-to solution since it’s the easiest and most convenient option… Especially during final exam periods when the pressure is high, I find myself particularly drawn to my phone.”* (P2)

*“My primary purpose for using my phone is browsing short video platforms, especially TikTok, which accounts for the largest proportion of my screen time.”* (P12)

#### 3.1.3 Negative affect

When medical students recognized their excessive phone use, they experienced significant guilt, understanding that their behavior was adversely affecting their studies and daily life, leading to regret and helplessness. Additionally, constant exposure to various information, social comparisons, and comments online often triggered anxiety related to academic performance and interpersonal relationships. The gap between their real-life circumstances and idealized online representations further intensified psychological pressure.

*“I’m still somewhat addicted to my phone, which leads to poor study efficiency and makes me feel frustrated… I also feel a bit guilty about it.”* (P6).

*“Whenever I feel stressed about studying for the civil service exam, seeing news about unemployment and job-hunting difficulties on my phone… Makes me anxious about my future career.”* (P7).

*“I feel very guilty, especially when I have not done anything meaningful for a long time and instead spend time playing (using my phone). I feel remorseful and have a sense of guilt, thinking I should not be like this.”* (P15).

### 3.2 Competence

#### 3.2.1 Sense of achievement

Victories and progress in mobile games provided medical students with immediate satisfaction and a sense of accomplishment. Similarly, mastering new skills through mobile phone applications or online learning platforms offered a sense of competence and satisfaction that was more immediate than their lengthy medical education process.

*“When using my phone, I occasionally play games, and each time I complete a level or beat a game, I experience a sense of accomplishment. Additionally, I enjoy reading novels and finishing a story brings me a powerful feeling of satisfaction, providing significant psychological pleasure and a genuine sense of achievement.”* (P3).

*“When I play games, achieving certain goals in these virtual worlds gives me a sense of accomplishment and helps relieve my stress.”* (P5).

*“During the game, at the moment of victory, I feel a great sense of achievement.”* (P12).

*“Take getting a certificate photo, for example. I used to think I had to go to a photo studio to get it taken. Later, I discovered I could actually do it myself with my phone. It feels pretty good, you know. Just learning something new gives you a little sense of achievement.”* (P13).

*“Winning a game gives me a strong sense of accomplishment… and I feel really happy when I achieve certain ranks or levels in the game.”* (P15).

#### 3.2.2 Social competence impairment

Extended reliance on mobile phones for communication diminished opportunities for face-to-face interactions, resulting in deteriorating real-world social skills. Participants noted that their verbal communication abilities, including language organization and expression, had weakened due to their preference for digital communication.

*“When making friends and chatting online, digital emojis and stickers can better express what I want to convey. However, in real life, my social and communication skills have declined somewhat…”* (P6).

*“Through internet platforms, I can connect with many students I did not know before. I can actively join groups of different majors or engage in exchanges with students who are knowledgeable in fields I’m not familiar with. This approach provides me with valuable opportunities for cross-disciplinary and cross-field learning and communication, helping me expand my knowledge horizons and promote a diverse learning experience.”* (P8).

*“I’ve noticed that excessive mobile phone use might impair my language expression skills. Frequently relying on mobile devices for communication has weakened my verbal communication skills and ability to interact face-to-face.”* (P9).

*“When using my phone for online socializing and chatting, I communicate more freely and confidently. However, in face-to-face interactions, I often find myself at a loss for words.”* (P13).

*“In real life, I rarely engage in deep conversations with classmates, often feeling tense and uncomfortable; in contrast, I can express myself freely online. Virtual communication does not require face-to-face interaction, eliminating the pressure of direct eye contact and reducing feelings of social fatigue and shyness.”* (P15).

### 3.3 Relatedness

#### 3.3.1 Social relationship maintenance

Given geographic separation and busy schedules, mobile phones have become essential tools for maintaining existing relationships. Medical students regularly use their phones to stay connected with friends and family, share personal experiences, and strengthen emotional bonds. They also established and maintained connections with course instructors and mentors via mobile platforms, consulting teachers about academic questions outside of formal class time.

*“Besides friends, I can meet in person, most of my other friends are from the past, and since we are not in the same place anymore, we can only stay in touch through our phones… I also occasionally use my phone to ask teachers questions.”* (P1).

*“My phone has been a great help with my social connections. It allows me to stay in touch with classmates during holidays and maintain relationships with old friends I have not seen for years… I can also add teachers on WeChat, which makes it more convenient to communicate with them and get more information.”* (P5).

*“After starting university, with old friends now separated, my phone has become a way to maintain emotional connections. It’s also convenient for staying in touch with family and sharing updates about my life… I regularly use it to ask teachers questions about my studies.”* (P15).

#### 3.3.2 Digital medical identity formation

Medical students engaged in information exchange through various platforms with members of their online communities, receiving increased social support through these digital networks. They built new medical professional interpersonal networks through social media and specialized forums, which served to compensate for limitations in face-to-face interactions and expand their social circles beyond geographic constraints.

*“I usually use my mobile phone to search for knowledge related to my professional field. When encountering difficult problems in my studies, I actively seek solutions online, especially those instructional videos with practical demonstrations.”* (P1).

*“I joined various group chats for activities and department recruitment, where I met many outstanding students. Through subsequent interactions, some of these classmates gradually became close friends.”* (P5).

*“With the continuous development of the internet, I can now access professional knowledge that was previously difficult to obtain in real life. Especially in fields like medicine and nursing, the network provides me with information and resources crucial for my future career development and planning. Through these platforms, I can better understand industry dynamics and professional development trends, helping me better plan and prepare for my career path.”* (P8).

*“My phone helps expand my virtual social network – through joining college group chats or private conversations, I can better understand information about different majors.”* (P15).

*“During our diagnostic medicine course, we utilized a clinical simulation platform on our smartphones to practice virtual case diagnosis. This interactive learning approach helped us systematically understand the disease diagnostic process.”* (P9).

### 3.4 Meaning in life

#### 3.4.1 Life direction confusion

Medical students reported profound confusion regarding their life goals and direction, manifesting as feelings of emptiness and lack of clear life purpose. Participants described experiencing a pervasive sense of meaninglessness in their daily lives, questioning the fundamental reasons behind their studies and existence. Many expressed uncertainty about their future career paths, leading them to seek temporary relief through mobile phone use as a form of cognitive avoidance.

*“My daily life is just attending classes, playing with my phone, and sleeping… During my free time, I do not know what to do, so I just scroll through my phone and watch videos, and time passes like that. Sometimes I suddenly ask myself, what’s the point of living like this? I feel that every day is repetitive, nothing special, and my heart always feels empty.”* (P1).

*“I feel my life is monotonous and empty… I often ask myself what’s the meaning of living like this, but I cannot find an answer. My phone seems to be the only way to fill this emptiness, but after scrolling through it, the feeling of emptiness becomes even stronger.”* (P3).

*“Life feels like I’m just going through the motions without any real direction or purpose. Whenever I feel that emptiness inside, I reach for my phone as a way to escape from the discomfort.”* (P5).

*“My parents initially encouraged me to study medicine, saying this profession is stable with high social status. But now I really do not know what I want… I feel like I’m being pushed forward, but I’m not clear about what lies ahead.”* (P9).

*“To be honest, I’m not quite sure why I’m studying medicine, and I do not know what kind of person I want to become in the future. Daily studying feels like just completing tasks, without any clear goals or direction… I’m actually very confused about the future.”* (P10).

#### 3.4.2 Value confusion

Medical students exhibited confusion regarding values and spiritual pursuits, lacking clear value standards and spiritual anchoring. Participants expressed uncertainty about what constituted truly important and valuable aspects of life, struggling to establish a coherent personal value system. This value disorientation manifested as difficulty distinguishing between meaningful and trivial matters, creating anxiety that prompted escape through mobile phone use. The constant exposure to diverse value systems through social media further exacerbated their confusion.

*“I feel that my spiritual world is very impoverished, with nothing that can truly make me feel fulfilled and satisfied in my pursuits. I see some classmates who have very clear ideals and beliefs, and I envy them, but I cannot find that kind of spiritual strength to support my progress.”* (P6).

*“I have been searching for what is truly important in life. I try to find it through my phone, but I can never find it.”* (P13).

*“What confuses me most is that I find myself without clear attitudes toward many things. I always waver between* var*ious viewpoints and cannot find values that I truly identify with.”* (P15).

*“Seeing various viewpoints and values online, I feel very confused. Sometimes I think this makes sense, sometimes I think that is right, but I have no idea what is truly correct.”* (P8).

## 4 Discussion

### 4.1 Principal finding

Based on the BPNT, our study reveals that the mobile phone dependence of medical students’ behaviors stems from three unmet psychological needs, including autonomy, competence, and relatedness, while also revealing meaning in life that extends beyond the BPNT framework. Among these, relatedness is the primary factor. We uniquely discovered that medical students use mobile phones for reality pressure evasion to meet their autonomy, pursue a sense of achievement to meet their competence, engage in medical digital social networking to meet their relatedness and seek external meaning compensation through smartphones to meet their meaning in life.

Among the driving factors of mobile phone dependence behaviors of medical students, unmet autonomy needs manifest in three aspects, which include low self-regulation, reality pressure evasion, and negative affect. Our study has found that medical students with mobile phone dependence commonly exhibit low self-regulation ability and negative affect consistent with previous studies showing that loneliness leads to smartphone addiction through impaired self-regulation (Gökçearslan et al., 2016), poor self-efficacy increases addiction risk (Mahapatra, 2019), problematic mobile phone use shows strong bidirectional associations with negative affect states such as anxiety and depression (Elhai et al., 2017; Wolniewicz et al., 2018). Notably, our study found that reality pressure evasion primarily drives medical students’ mobile phone dependency behaviors, which represents their attempt to address unmet needs for autonomy. Medical students often seek temporary spaces for rest to maintain psychological balance under high-intensity academic pressure, which aligns with the autonomy need hypothesis in the BPNT that suggests individuals naturally desire a sense of freedom and relief from external pressure (Deci and Ryan, 2000; Vansteenkiste et al., 2020; Ryan and Deci, 2000). We found that mobile phone dependence among medical students is not merely a simple reality of pressure evasion but reflects an unmet need for autonomy underlying this behavior. Mobile phones become a psychological buffer zone in high-intensity learning environments, allowing medical students to temporarily escape feelings of external control (Kardefelt-Winther, 2014) and regain brief experiences of autonomy, which suggests that the use of mobile phones is an actively constructed psychological regulation approach by medical students under extreme pressure, although this may lead to phone dependence.

Unmet competence needs primarily manifest as a sense of achievement and social competence impairment. Our study found an association between mobile phone dependence and diminished social performance, similar to previous studies that indicate mobile phone dependence impairs medical students’ communication skills (Celikkalp et al., 2020) and disrupts attention and social interaction through frequent notifications (Kushlev et al., 2016). Our study uniquely found that the mobile phone dependence behavior of medical students partially stems from their need to obtain a sense of achievement. Mobile phones provide students with an alternative means of obtaining a sense of achievement, which aligns with the BPNT, emphasizing that the need for competence reflects an individual’s intrinsic motivation to demonstrate and develop skills (Deci and Ryan, 2000; Vansteenkiste et al., 2020; Ryan and Deci, 2000). However, medical students must undergo years of theoretical learning before gaining opportunities for actual clinical practice and validation of their professional abilities (Bugaj et al., 2016). This delayed gratification of competence needs drives medical students to turn to mobile phones for an immediate and fleeting sense of achievement.

Interestingly, unmet relatedness needs, including social maintenance and medical digital social networking, play a dominant role in the mobile phone dependence of medical students. Our study found that medical students commonly use mobile phones to maintain various social relationships across spatial limitations, consistent with previous studies that show mobile phones help students maintain connections with family, friends, and teachers while adapting to new environments, allowing people to sustain these diverse social bonds through frequent, brief communications even when physically separated (Chen, 2007; Wajcman et al., 2008; Licoppe, 2004). Additionally, our study uniquely found that medical digital social networking is another key factor in the mobile phone dependence of medical students, aligning with the BPNT, which states that relatedness needs to manifest as a fundamental desire to establish meaningful social connections and a sense of belonging (Deci and Ryan, 2000; Vansteenkiste et al., 2020; Ryan and Deci, 2000). Medical students use their mobile phones to access and share professional information, discuss career challenges, develop professional perspectives, build online professional networks gradually, and begin to develop their professional identities even before formally entering the healthcare field, which suggests that their reliance on mobile phones should not be simply categorized as problematic behavior, but rather understood as an adaptive mechanism for navigating the challenges of professional identity formation. Although there have been similar studies previously (Cruess et al., 2018), they did not consider the factor of unmet relatedness needs. Notably, our study found that relatedness needs play a dominant role in driving mobile phone dependence of medical students, which differs from traditional psychological models that simply attribute dependent behaviors to pleasure-seeking or discomfort-avoidance (Baker et al., 2004; Volkow and Morales, 2015).

Notably, our study unexpectedly identified meaning in life as a psychological need underlying smartphone dependence among medical students when exploring this phenomenon within the BPNT framework, representing an important finding beyond the theoretical scope. Meaning in life refers to the degree to which individuals perceive their existence as valuable, reflecting their intrinsic experience of seeking life direction and purpose, and as a positive psychological resource, meaning in life importantly impacts both physical and mental health (Frankl, 1985). A previous cross-sectional study found that individuals with a strong sense of meaning in life better recognize their existential purpose and mission, pursuing clear life goals and long-term values. These individuals are more likely to possess clear life direction and effectively avoid boredom and emptiness (Chen et al., 2025). A longitudinal study confirmed that individuals with weaker meaning in life are more susceptible to smartphone addiction, and importantly, revealed the reverse relationship whereby smartphone addiction diminishes one’s sense of meaning in life (Zhao et al., 2023). Although our findings align with previous studies, we provide deeper insights into how medical students’ lack of meaning in life manifests as intertwined life direction and value confusion. Medical students face dual pressures of upholding traditional medical profession ideals while confronting contemporary value pluralization, leading to existential questions about their purpose and goals. When mobile phones expose them to diverse values, students lacking clear internal standards experience heightened confusion and anxiety. A previous study has demonstrated that medical students face fundamental conflicts about what values they should bring to their profession, namely individual values or professional ones, and this conflict is further exacerbated when they are exposed to multiple value systems through digital media (Park and Hong, 2022). In this meaning-deficit state, mobile phone use becomes a compensatory mechanism, but such external meaning-seeking cannot replace authentic self-direction recognition, often resulting in deeper emptiness and dependency.

### 4.2 Recommendations

In terms of unmet autonomy needs, our study found that medical students frequently use mobile phones to escape reality pressure in search of autonomy. Autonomous-supportive teaching methods have also been proven to significantly enhance medical students’ intrinsic motivation (Orsini et al., 2015). Accordingly, we recommend that medical schools should optimize curriculum arrangements, offer more elective learning tasks, and redesign assessment systems to create greater autonomy for students, helping them avoid seeking a sense of autonomy through smartphone use as an escape from pressure. Regarding unmet competence need, our findings show that medical students seek immediate achievement through mobile games and applications. Meanwhile, workplace curricula based on Entrustable Professional Activities (EPAs) have been proven effective in assessing actual clinical competence (ten Cate et al., 2010). Therefore, we recommend that medical schools establish EPA-based assessment systems that integrate theoretical learning with specific clinical tasks, providing students with clear competence progression pathways from “execution under supervision” to “independent completion” and ultimately to “ability to guide others.” This structured competence validation approach can effectively replace the fragmented sense of achievement that students currently obtain through smartphone use. Our study found that medical students lack capability validation during lengthy theoretical learning phases. A previous study has shown that students who received narrative-based virtual patient training demonstrated significantly better overall communication skills after 1 week compared to those who received problem-solving training (Bearman et al., 2001). Based on this empirical evidence, we recommend developing narrative-based virtual medical simulation platforms that allow students to validate their professional competencies during theoretical learning phases through immersive doctor-patient interaction experiences. These simulated clinical scenarios not only enhance technical proficiency but simultaneously promote professional confidence and identity formation through achievement experiences. Concerning unmet relatedness need, our study found that relatedness needs are the dominant factor in medical students’ mobile phone dependence, particularly manifested as needs for medical digital social networking. Various formal mentoring models for medical students and doctors have been confirmed to promote medical students’ professional development (Buddeberg-Fischer and Herta, 2006). Based on this empirical research, we recommend that medical schools establish structured mentoring systems and thoughtfully integrate digital professional socialization into these frameworks. By combining professional medical online social platforms with mentoring systems, medical schools can cultivate students’ digital professionalism while simultaneously providing healthy social channels to meet relatedness needs, thereby reducing dependence on non-professional social media.

Additionally, professional interventions from school psychologists and counselors are equally indispensable. On one hand, our study found that medical students with low self-regulation abilities and lacking meaning in life tend to use mobile phones to escape from real-life pressures and seek external meaning compensation, which paradoxically deepens their sense of emptiness and dependency. Notably, mindfulness training based on cognitive behavioral therapy has been shown to reduce smartphone usage time and addiction levels among college students (Lan et al., 2018), while logotherapy-based mindfulness intervention has been demonstrated to effectively reduce internet addiction levels among adolescents (Liu et al., 2021). Therefore, our study suggests school psychologists can conduct group mindfulness training courses based on logotherapy and cognitive behavioral therapy to help students discover and construct personal meaning in life, improve self-regulation abilities, provide cognitive restructuring technique training to help students identify and change irrational cognitions related to mobile phone use, and design targeted stress management programs to offer students healthy alternative methods for stress relief. On the other hand, our research has found that medical students’ mobile phone dependence behaviors are partly due to low self-regulation ability and reality pressure evasion, with unmet relatedness need being a key factor in their phone dependence. Group-based intervention methods have been proven to effectively improve individual self-regulation abilities, while professional online social platforms can cultivate users’ digital professionalism while meeting their social needs (Ko et al., 2015). Based on these findings, we recommend that academic advisors organize students into mutual support groups, encouraging them to set goals together and share their progress. Establishing professional online communication platforms can guide students to use digital media appropriately, reduce dependence on non-professional social media, and provide a safe environment for students to express opinions and receive support.

### 4.3 Limitations and future directions

This study has several limitations. First, our study sample was limited to medical students from a single medical university, potentially making the findings inapplicable to medical students from different regions or non-medical students. Future studies should extend to medical student populations across various geographical areas. Second, there is a gender imbalance issue in our study, and future studies should employ sampling strategies to ensure a more balanced gender ratio. Third, our data collection relied primarily on interviews, which introduces a strong subjective element and lacks objective measurements. Future studies should combine quantitative questionnaires with multi-dimensional assessments to capture the complexity of smartphone dependency among medical students more objectively. Finally, our study designed interview questions based on the three core concepts of BPNT, which may have influenced participants’ responses to some extent. Although we allowed themes to emerge before comparing them with the theoretical framework during data analysis to reduce the influence of theoretical presuppositions, future studies could incorporate quantitative validation methods to provide a more comprehensive understanding.

## 5 Conclusion

Our study shows that mobile phone dependence among undergraduate medical students reflects three unmet basic psychological needs, including autonomy, competence, and relatedness, and an additional meaning in life that transcends the theoretical framework. Relatedness remains the dominant factor among the BPNT needs. Specifically, facing intense reality pressure, medical students often use mobile phones to seek temporary spaces for rest to meet their need for autonomy; meanwhile, the delayed feedback in professional learning leads them to turn to mobile activities for an immediate sense of achievement, meeting their need for competence; furthermore, they utilize medical digital social platforms to establish professional connections and identity formation, meeting their need for relatedness. Our study recommends that medical schools should provide an autonomy-supportive teaching environment through optimized curriculum arrangements and redesigned assessment systems, create authentic opportunities for professional skill verification through EPA-based assessments and narrative-based virtual simulations, and establish structured mentoring systems with integrated digital social platforms that promote professional identity development. Simultaneously, evidence-based interventions from school psychologists and counselors, such as mindfulness training courses based on logotherapy and cognitive behavioral therapy and group-based self-regulation programs, are essential for cultivating healthy mobile phone usage habits among medical students.

## Data Availability

The raw data supporting the conclusions of this article will be made available by the authors, without undue reservation.
